# Edge-Aware Unidirectional Total Variation Model for Stripe Non-Uniformity Correction

**DOI:** 10.3390/s18041164

**Published:** 2018-04-11

**Authors:** Ayoub Boutemedjet, Chenwei Deng, Baojun Zhao

**Affiliations:** School of Information and Electronics, Beijing Institute of Technology, Beijing 100081, China; bout.ayoub@bit.edu.cn (A.B.); zbj@bit.edu.cn (B.Z.)

**Keywords:** Edge-aware weighting, stripe non-uniformity, total variation

## Abstract

The problem of stripe non-uniformity in array-based infrared imaging systems has been the focus of many research studies. Among the proposed correction techniques, total variation models have been proven to significantly reduce the effect of this type of noise on the captured image. However, they also cause the loss of some image details and textures due to over-smoothing effect. In this paper, a correction scheme is proposed based on unidirectional variation model to exploit the direction characteristic of the stripe noise, in which an edge-aware weighting is incorporated to convey image structure retaining ability to the overall algorithm. Moreover, a statistical-based regularization is also introduced to further enhance correction performance around strong edges. The proposed approach is thoroughly scrutinized and compared to the state-of-the-art de-striping techniques using real stripe non-uniform images. Results demonstrate a significant improvement in edge preservation with better correction performance.

## 1. Introduction

Spatial non-uniformity continues to represent a major downside in Focal Plane Arrays (FPA)—based infrared imaging systems. Non-uniformity is observed when the response of different array detectors to the same scene is different [1], such phenomenon creates an undesirable and time-dependent fixed pattern noise (FPN) imposed on the raw image which degrades its quality and undermines the performance of the imaging system. Hence, non-uniformity correction (NUC) that compensates for this undesirable noise has to be conducted before any other process can be efficiently performed on the captured image.

Many correction methods were reported and widely used which can be categorized into two main approaches, namely: calibration-based non-uniformity correction CBNUC [1,2] and scene-based non-uniformity correction SBNUC [3,4,5]. Traditional one-point and two-point methods that rely on uniform reference sources to extract and eliminate the non-uniform pattern fall in the class of calibration-based techniques. On the other hand, methods that exploit scene information are considered as scene-based approaches. In this category, we can find statistical approaches [3] and registration-based methods [4,5] both considered as classical techniques in the field of non-uniformity correction.

One type of the fixed pattern noise that commonly appears in infrared images is the stripe non-uniformity. It is mainly caused by the fact that detectors in the same column (respectively line) share one amplifier leading to vertical (respectively horizontal) grid-like lines to be forced upon the image scene, which makes the content of the image unrecognizable and difficult to exploit. Previously discussed traditional NUC techniques are not suitable for this kind of FPN and can find difficulties to correct for it [6]. However, other methods that deal specifically with stripe non-uniformity have been proposed. These de-striping approaches can be assigned to different categories. The first category consists of methods that exploit data and noise characteristics observed in the array. For instance, Tendero et al. [7] assumed that detectors in adjacent columns observe the same range of data implying that their histograms should be nearly equal, otherwise they are mapped to match a fixed reference histogram. Cao et al. [6,8] studied the relationship between the stripe noise and the scene data and derived a polynomial model that they used to distinguish between edges and textures that belong to the scene and the actual stripe FPN. Chang et al. [9] proposed to treat the destriping problem as a decomposition task using both the low-rank constraint, that exploits characteristics of the stripe noise, and the spectral information of the remote sensing images. Liu et al. [10] also proposed a method that separate stripe noise from the image using three constraints based on properties of the noise, namely: the sparsity, smoothness and discontinuity. The second category covers approaches that engage the stripe non-uniformity problem in the frequency domain, benefiting from the periodic nature of stripes it applies an adequate filter to remove them [11]. Recently, a new category was prposed by Kuang et al. [12] based on the exploitation of deep convolutional networks, where they used both image denoising and super resolution to eliminate the stripe noise in the input and produce a clear image at the output with well-preserved edges. Finally, the third category and most relevant to the present work is the optimization-based approach [13,14,15], where the correction process aims to estimate the corrected image by minimizing a cost function that mutually ensures the correction for stripe FPN along with the preservation of image details. Under these methods the cost function is usually formed by two terms, the first one responsible for removing the stripe noise called the “regularization term” and the second one helps to preserve the detail information during the correction called the “fidelity term”.

All approaches mentioned above trade-off in their process between two major goals, reducing the effect of the FPN on the image and retaining image texture and details. The effort to find a balance between these two objectives represents the main challenge of these methods. In the case of total variation techniques, the focus has been turned to the regularization term where a mechanism to distinguish between stripe noise and image edges is crucial for achieving the aforementioned goals. Zhao et al. [13] proposed a gradient-constrained approach where the gradient along the stripe direction is preserved while the energy of the one along the opposite direction is minimized. Chang et al. [14] proposed a two-part regularization term, the first part puts constraints on gradients both across the stripe lines to remove them and along the stripe lines to preserve detail information, in addition to a second part that uses the framelet regularization to preserve structural details that the first part cannot properly retain. Huang et al. [15] proposed a unidirectional variational model optimization method that uses iteratively reweighted least square technique, their method provides an automatic formula to appropriately update the regularization parameter in order to achieve efficient correction.

Based on previous work on variation model correction, the proposed work consists of an improved unidirectional version that eliminates stripe noise by penalizing the gradient across the stripe direction under the guidance of an edge-aware weighting matrix. The weighting values are attributed according to the nature of the pixel in the image structure. A regularization operation is also applied to the estimated stripe FPN to preserve strong edges that may still be smoothed after the correction. In this regularization, separation between edges and noise is made based on assumptions on the noise statistical model, hence the name statistical regularization.

This paper is organized as follows. Section 2 is dedicated to a detailed description of the proposed algorithm. Then, experimental results are presented along with discussions in Section 3 followed by conclusions in Section 4 to sum up the presented work.

## 2. Experimental Details

### 2.1. Total Variation Optimization

**Figure 1:**
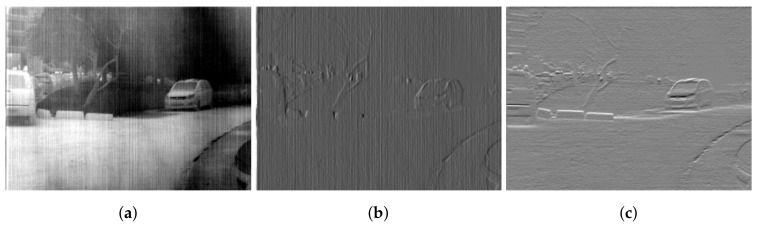
Directional characteristic of the stripe FPN (**a**) noisy image (**b**) Vertical gradient (**c**) Horizontal gradient.

Stripe non-uniformity is usually modeled as an additive noise, which allows us to represent the non-uniformity of an infrared image as follow:

(1) f(x,y)=u(x,y)+n(x,y),

where f(x,y) is the observed noisy image, u(x,y) is the clean image and n(x,y) is the stripe FPN. Total variation approach for non-uniformity correction falls in the category of solving an inverse problem where the estimated true scene is deduced from the degraded image. Moreover, this problem is ill-posed and requires additional information that helps put regularizing constraints on the solution to obtain satisfying results. Stripe structure is a well-known propriety of the stripe noise, which is widely used as prior information. Degradation is more severe in the horizontal direction (assuming that stripes are vertical) than in the vertical direction, such behavior can be observed using the horizontal and vertical gradients of a corrupted image as depicted in Figure 1. We can clearly see that the horizontal gradient suffers from considerable variations while the vertical gradient is hardly affected by the stripe noise.

Hence, for a correction scheme that involves variational model it is intuitive to set the fidelity term as to sustain the vertical gradient and set the regularization term as to penalize the energy of the horizontal gradient. Such scheme is best described using the following energy function of a unidirectional variational model:

(2) $$E\left(u\right)=\frac{1}{2}{∥\nabla_{y}\left(u-f\right)∥}_{1}+\lambda {∥\nabla_{x}\left(u\right)∥}_{1},$$

where ${∥.∥}_{1}$ represents the $l_{1}$-norm, $\nabla_{x}$ and $\nabla_{y}$ refers to the horizontal and vertical gradient operator respectively and λ is a regularization parameter that controls the smoothness of the corrected image. The motivation behind choosing the $l_{1}$-norm comes from its better performance in edge preserving [16].

Minimizing the energy function in Equation (2) as it is cannot produce a satisfactory solution to the de-striping problem. Setting the same regularization parameter for the whole image is unreasonable given the different features present in it. In other words, weights assigned to stripe noise should not be the same as the one assigned to an image edge otherwise the solution will be over-smoothed and detail information will be lost. One way to overcome this issue is to assign a weighting matrix to the regularization term where: high-value weights are attributed to regions where the stripe noise is important to eliminate it, while small value weights are chosen for regions containing image textures and details to avoid smoothing them. Hence, the new cost function will have the following form:

(3) $$E\left(u\right)=\frac{1}{2}{∥\nabla_{y}\left(u-f\right)∥}_{1}+\lambda D_{f}{∥\nabla_{x}\left(u\right)∥}_{1},$$

where $D_{f}$ is the weighting matrix containing per-pixel weights that control the amount of influence the horizontal gradient constraint should have on the final image. Its computation is explained in the next section. The optimization of Equation (3) presents some difficulties, mainly the non-differentiability of the $l_{1}$-norm. Many solutions have been proposed to deal with this problem, for instance the Split Bregman iteration [17] and the iterative reweighted least squares (IRLS) method [18]. In our work, we opted for the latter for its computational efficiency and flexibility. Under the IRLS algorithm the variational functional in Equation (3) is approximated as follow:

(4) $$\tilde{E}\left(u\right)=\frac{1}{2}{∥W_{1}^{1/2}\left(\nabla_{y}\left(u-f\right)\right)∥}_{2}^{2}+\frac{\lambda }{2}D_{f}{∥W_{2}^{1/2}\nabla_{x}\left(u\right)∥}_{2}^{2},$$

where $W_{1}=diag\left(2\phi \left(\nabla_{y}\left(u-f\right)\right)\right)$, $W_{2}=diag\left(2\Psi \left(\nabla_{x}\left(u\right)\right)\right)$. The notation diag(v) refers to a diagonal matrix of the vector *v*, ϕ(v) and Ψ(v) are given as:

(5) $$\phi \left(v\right)=\left\{\begin{array}{cc} {\left|v\right|}^{-1}, & |v|>\epsilon_{1},\\ \epsilon_{1}^{-1}, & \left|v\right|\leq \epsilon_{1}, \end{array}\right.,\Psi \left(v\right)=\left\{\begin{array}{cc} {\left|v\right|}^{-1}, & |v|>\epsilon_{2},\\ \epsilon_{2}^{-1}, & \left|v\right|\leq \epsilon_{2}, \end{array}\right.$$

where $\epsilon_{1}$ and $\epsilon_{2}$ are small positive numbers chosen to avoid division by zero-valued components. The new cost function $\tilde{E}\left(u\right)$ gradient is evaluated as follow:

(6) $${\tilde{E}}^{\prime }\left(u\right)={\left(\nabla_{y}\right)}^{T}W_{1}\left(\nabla_{y}\left(u-f\right)\right)+\lambda D_{f}{\left(\nabla_{x}\right)}^{T}W_{2}\nabla_{x}\left(u\right).$$

Finally, a gradient descent scheme is used to update the solution:

(7) $$u_{n+1}=u_{n}-\Delta t{\tilde{E}}^{\prime }\left(u\right).$$

where Δt is the convergence step. The iterative solving process is halted under the following condition: $|u_{n+1}-u_{n}|\leq tol$, where tol is the tolerance parameter.

### 2.2. Edge-Aware Weighting

In light of the above discussion, the weight matrix of the regularization term must control the penalization of stripe noise in order to preserve image textures. In other words, the weight matrix have to efficiently extract detail information and separate them from noise structure present in the image. To carry out this task, a well-known edge-aware weighting is adopted for a total variation approach to correct stripe FPN.

**Figure 2:**
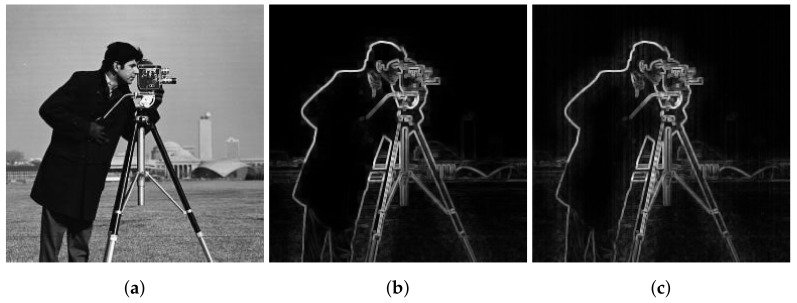
Comparison of the edge-aware weighting in case of clear and noisy image (**a**) Input image (**b**) Case of clear image (**c**) Case of noisy image.

Inspired by previous work on gradient domain optimization and edge-aware constraints [19,20], an explicit weighting $\Gamma_{f}\left(p\right)$ that efficiently describes image edges is defined. It is computed using local variances of 3 × 3 and *r* × *r* windows of all image pixels in the image *f*:

(8) $$\Gamma_{f}\left(p\right)=\frac{1}{N}\sum_{i=1}^{N}\frac{\sigma_{f,3}\left(p\right)\sigma_{f,r}\left(p\right)+\epsilon }{\sigma_{f,3}\left(i\right)\sigma_{f,r}\left(i\right)+\epsilon },$$

where $\sigma_{f,3}\left(p\right)$ and $\sigma_{f,r}\left(p\right)$ are the standard deviation of image *f* in a 3 × 3 window and an *r* × *r* window (*r* represents the window size) respectively both centered at the pixel *p*, *N* is the number of pixels in the image *f* and ϵ is a small positive constant value that is usually selected as ${\left(0.001\;\times \;L\right)}^{2}$ where *L* is the dynamic range of the image. From the definition of $\Gamma_{f}\left(p\right)$ we can deduce that its role is to measure the importance of a given pixel *p* with respect to the whole image *f*. Furthermore, it uses a small scale in addition to a larger scale which helps to efficiently separate edges from fine details and enhances the performance of the weighting factor. Figure 2b shows the ability of the weighting $\Gamma_{f}\left(p\right)$ to depict image edges and details with accuracy. However, in case of presence of stripe noise in the image, the weighting will wrongly attribute some noise structure (vertical stripes) to the image edges as seen in Figure 2c. To overcome this problem, we propose to first separate the image smooth part $f_{s}$ from the high frequency part $f_{d}$ using horizontal filtering and then use both parts to set the weighting $\Gamma_{f}\left(p\right)$. The new weighting formula becomes as follow:

(9) $$\Gamma_{f}\left(p\right)=\frac{1}{N}\sum_{i=1}^{N}\frac{\sigma_{f_{s},3}\left(p\right)\sigma_{f_{d},r}\left(p\right)+\epsilon }{\sigma_{f_{s},3}\left(i\right)\sigma_{f_{d},r}\left(i\right)+\epsilon },$$

The new weighting will play the role of detecting similar structures in both image parts. We will further discuss this in the next section.

Finally the weighting matrix $D_{f}$ used in the variational model can be specified as follow:

(10) $$D_{f}\left(p\right)=\left\{\begin{array}{cc} 1, & if\;\Gamma_{f}\left(p\right)<S\\ \delta , & if\;\Gamma_{f}\left(p\right)\geq S \end{array}\right.$$

where $D_{f}\left(p\right)$ is the edge-aware matrix value at pixel *p*, *S* is a threshold to separate edges from smooth regions and δ is a small positive value attributed to weights around edges. Both parameter values of *S* and δ are chosen experimentally to ensure better performance. As it can be easily deduced from Equation (10), the edge-aware matrix attributes smaller weights to pixels that belong to edges and image structure in order to preserve them.

### 2.3. Horizontal Filtering

The purpose here is to separate the image into a smooth part completely free from stripe non-uniformity and a detail part that contains both the removed noise and some image textures blurred by the filtering process, then use both parts to construct the edge-aware weight matrix. Many state-of-the-art de-noising filters can be used to extract the high-frequency part of a noisy image without over-smoothing the details [21,22], however, the guided filter offers the highest efficiency in edge-preserving and structure transferring which fits the main objective to retain as many details as possible in the filtered image. Under the 1D guided filter, the noisy image *f* is filtered under the guidance of an image *g*. Following a local linear model, the smoothed image $f_{s}$ can be expressed as a linear transform of the guidance image *g* in a 1D row window $w_{k}$:

(11) $$f_{s}\left(i\right)=a_{k}g\left(i\right)+b_{k},\forall i\in w_{k}$$

where $a_{k}$ and $b_{k}$ are constant coefficients in the window $w_{k}$. These coefficients are computed by minimizing the mean square error between the filtered image and the noisy input *f* for each window $w_{k}$ under some regularization as follow:

(12) $$E\left(a_{k},b_{k}\right)=\sum_{i\in w_{k}}\left({\left(a_{k}g\left(i\right)+b_{k}-f\left(i\right)\right)}^{2}+\xi a_{k}^{2}\right),$$

where ξ is a regularization term to penalize large values of $a_{k}$. In the present work, the input image *f* is used as a guidance image and the regularization ξ is set to a high value in order to completely remove the stripe structure, as for $w_{k}$ a 1 × 9 row window is chosen. The resulting image of this step $f_{s}$ is then subtracted from the noisy one *f* to obtain the detail layer $f_{d}$.

### 2.4. Statistical Regularization

Although the contribution of an edge-aware weighting to the texture preserving capacity of the variational model, some image details are still present in the estimated FPN noise. Hence the motivation for an additional step that further eliminates residual details smoothed by the correction process. To engage this problem, a set of statistical assumptions are first made on the stripe FPN noise:

1. The non-uniformity along the same column is modeled as an unknown random variable that follows a Gaussian distribution with a mean $\mu_{y}$ and a standard deviation $\sigma_{y}$,
2. Non-uniformity noise in different columns of the array are considered to be independent of each other.

**Figure 3:**
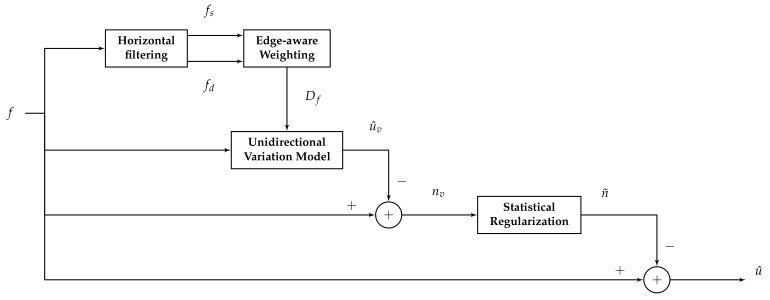
Scheme of the proposed method.

Following these two assumptions, the estimated stripe noise for each column *y* is inspected where any value that deviates from the distribution is eliminated as follow:

(13) $$\tilde{n}\left(x\right)=\left\{\begin{array}{cc} 0, & \left|n_{v}\left(x\right)-\mu_{y}\right|\geq 3\sigma_{y},\\ n_{v}\left(x\right), & \left|n_{v}\left(x\right)-\mu_{y}\right|<3\sigma_{y}, \end{array}\right.$$

where $n_{v}$ is the estimated noise obtained by subtracting the corrected image ${\hat{u}}_{v}$ estimated by the variational model from the the noisy image *f*. Finally, the regularized estimated FPN $\tilde{n}$ will be subtracted from the noisy image *f* to obtain the final corrected image. A complete scheme representing the whole correction process is presented in Figure 3.

## 3. Results and Discussion

To test the performance of the proposed edge-aware variational model, several experiments were conducted. First, the edge detection efficiency of the proposed weighting is validated, then the contribution of the statistical regularization to edge preserving is verified and finally the performance of the overall proposed algorithm is evaluated and compared to existing state-of-the-art correction methods.

### 3.1. Edge Preserving Performance

**Figure 4:**
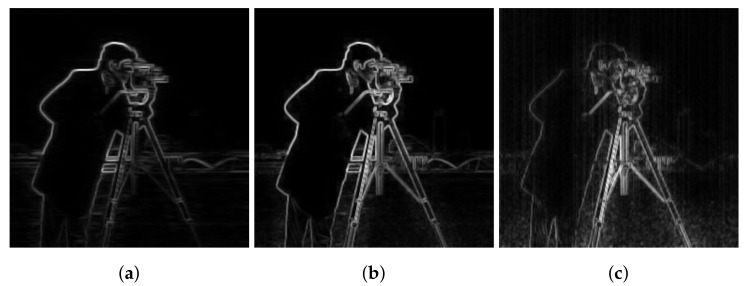
Comparison of the edge-aware weighting in case of smoothed part and detail part (**a**) Smoothed part used (**b**) The clear image used (**c**) Detail part used.

As mentioned in the previous section, the use of the noisy image to set the weighting will cause the appearance of stripes as part of the image structure that we seek to preserve. Hence the introduction of the horizontal filtering step to provide a smoothed noise-free version of the noisy image. However, a weighting based on this version will only depict edges that were not over smoothed by the horizontal filtering. In the same manner, using the high-frequency part will only show over smoothed edges along with some stripe FPN. Figure 4 shows the two cases where only the smoothed part or the detail part is used in the computing of the weighting $\Gamma_{f}$. For the sake of comparison, the clear image based weighting was computed to use it as a reference. In case of the smoothed image, it is seen that some edges belonging to the background buildings are missing, while vertical stripes are visible in the detail image case.

**Figure 5:**
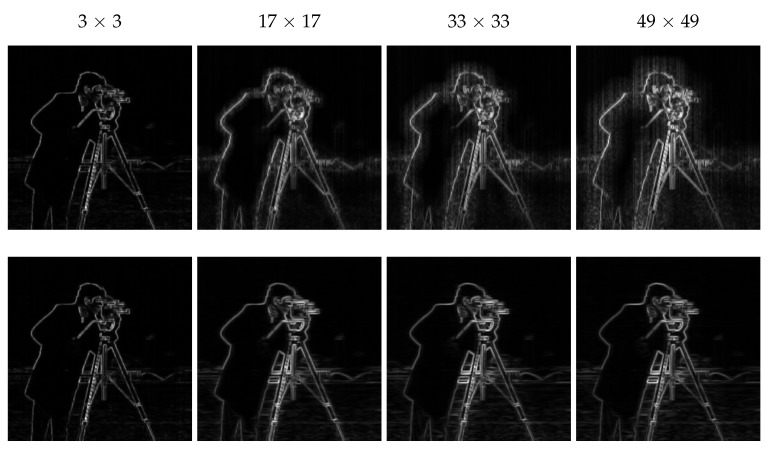
Different cases of setting the edge-aware weighting.

Therefore, in the proposed weighting, both the smoothed part and detail part are used to efficiently extract image structure from the stripe noise. Furthermore, experimental results show that using the small scale on the smoothed image and the large scale on the detail image along with setting the small window to a 3 × 3 window and the large one to 33 × 33 window (as suggested by Kou et al. [20]) provides better results. For instance, Figure 5 shows different possible combinations for setting the weighting factor.

On the first line, results are shown from the case where the small scale (3 × 3 window) is used on the detail part and the large scale on the smoothed part with different sizes of the window *r* × *r*, while the second line is dedicated to the inverse case. As it is clearly seen, the small scale is better used on the smoothed part to avoid the appearance of stripe FPN. As for the size of the large scale *r*, we find that the higher its value is the more details can be detected. However, starting from the value 33, enough image details are already detected to allow the edge-aware weighting to sufficiently represent image structure.

### 3.2. Validation of the Statistical Regularization

**Figure 6:**
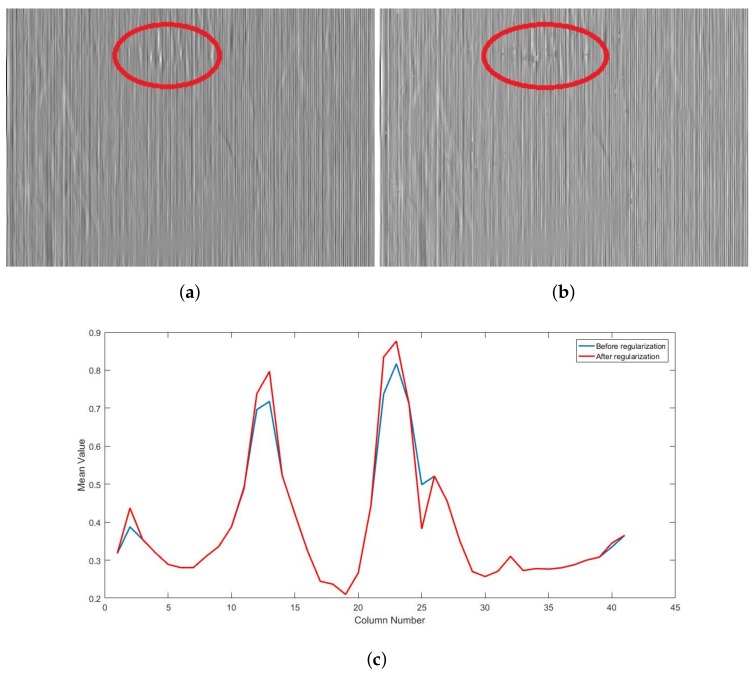
Efficiency of the statistical regularization (**a**) FPN before regularization (**b**) FPN after regularization (**c**) Mean cross-track profiles before (blue) and after (red) regularization.

The role of the statistical regularization is to preserve certain image details that are still present in the estimated non-uniformity image after the edge-aware correction. These details usually belong to regions of the image that are too bright or too dark compared to the whole image. Figure 6 shows the importance of this measure to further enhance the ability of the proposed algorithm to retain image structure after correction. Some bright edges that appear in the estimated noise (Figure 6a) can be clearly seen. These edges significantly differ from the noise, which make it easy for the statistical regularization to extract them and remove them from the estimated noise (Figure 6b). This can be done without affecting the correction, since these strong edges are usually not affected by the stripe FPN. The result is sharp and more accurate edges, as we can see in Figure 6c, where the mean cross-track profiles is computed for a 50 × 40 window, over a region where strong edges are present, in both cases before (blue) and after (red) regularization. In case of the later, edges are more sharp and well-defined.

### 3.3. Real Experiments

In order to validate the efficiency of the proposed algorithm, a comparison was made to three state-of-the-art methods, namely the midway infrared equalization (MIRE) [7], the 1D guided filtering (GIF) based method [6] and the iteratively reweighted unidirectional total variation model IRUTV [15]. Experiments were conducted on real stripe non-uniformity images obtained from a public dataset. Under the guidance of previous section, algorithm parameters are set as follow: λ=0.1, $\epsilon_{1}=0.0001$, $\epsilon_{2}=0.0001$, r=33, S=0.02, δ=0.2, Δt=0.1, ξ=0.1 and tol=0.0001. For the other methods, parameters are set according to what is recommended in their work.

#### 3.3.1. Qualitative Study

**Figure 7:**
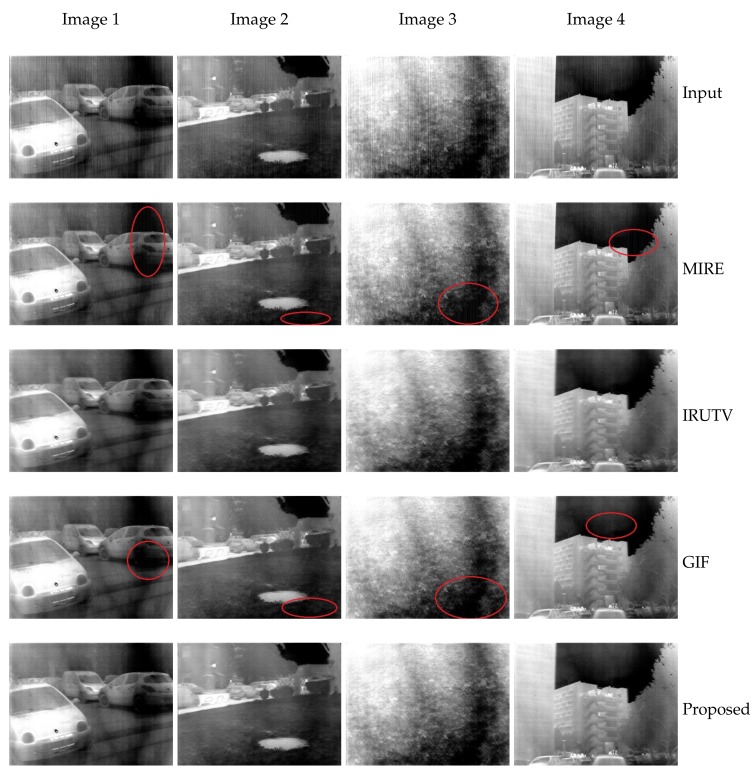
Comparative results of the proposed method and three de-striping techniques [6,7,15].

**Figure 8:**
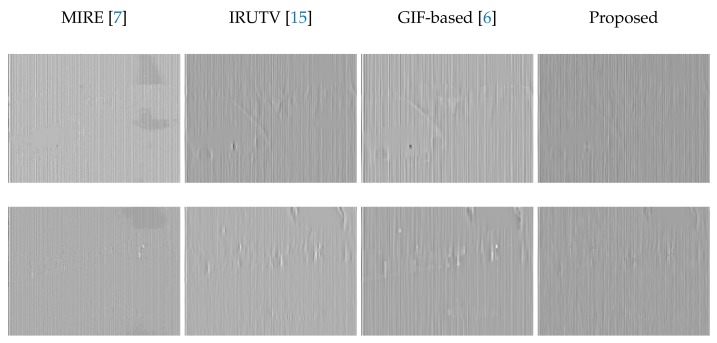
Estimated stripe FPN using the proposed method and three de-striping methods [6,7,15].

Figure 7 shows correction results for four images with different scene characteristics. The MIRE and guided filter based approaches both offer good edge preserving abilities but some uncorrected residual noise can be seen around edges (see the highlighted areas). The IRUTV however, exhibits a better noise elimination performance but it comes along with notable edge smoothing and detail blurring. In case of the proposed method, results show that it always outperform other methods providing smoothed and noise-free images with well-preserved edges and textures. The estimated stripe noise for the first two images are shown in Figure 8, some edges and features with varying levels were wrongly considered as FPN in the case of the three state-of-the-art algorithms. While in the case of the proposed method and due to the statistical regularization, the estimated noise image contains mainly the stripe FPN.

**Figure 9:**
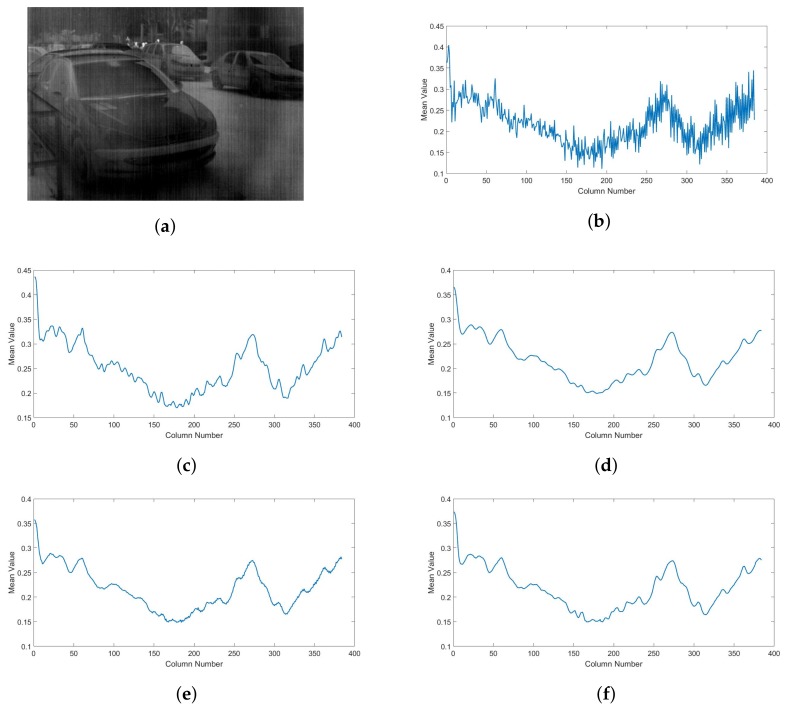
Mean cross-track profiles of a corrupted image and its correction versions: (**a**) Noisy image (**b**) Without correction (**c**) MIRE (**d**) IRUTV (**e**) GIF-based (**f**) Proposed.

To further prove these findings, the mean cross-track profiles is computed for a noisy image and its corrected versions using the four algorithms. Results are shown in Figure 9. The effect of stripe noise can be seen as rapid variation of the mean value from column to column as seen in Figure 9b. After the correction, the MIRE algorithm (Figure 9c) reduces these changes but some small fluctuations are still present which refers to residual non-uniformity that was not corrected. Same remark can be noticed in the case of GIF-based approach (Figure 9e). On the other hand, the IRUTV algorithm completely remove these variations however some variations that belongs to image textures are also smoothed and the mean cross-track appears to have an over-smoothed profile (Figure 9d). Meanwhile, the proposed algorithm efficiently smooths fluctuations belonging to the stripe noise with good preservation of the small changes corresponding to image details (Figure 9f).

Finally, the proposed algorithm was tested using image sequences from the work of Portmann et al. [23]. The results are depicted in video 1 and video 2, where the noisy version is presented (top-left corner) along with the correction results of the IRUTV algorithm (top-right corner), the GIF-based method (bottom-left corner) and the proposed approach (bottom-right corner). An over-smoothing effect can be noticed in the case of the IRUTV algorithm and some residual noise appearing in the case of the GIF-based method. Clearly the proposed algorithm offers better performance by ensuring the smooth and noise-free corrected image with sharp and well-preserved edges.

#### 3.3.2. Quantitative Study

In this part, an evaluation of the method is conducted using a quantitative measure such as the PSNR, which stands for the peak signal-to-noise ratio, and it is defined as follows:

(14) $$PSNR=20\times log_{10}\left(\frac{2^{b}-1}{rmse}\right),$$

where *b* is the number of bits that represents a pixel value in the image (8 bits in our case), rmse is the root mean square error between the estimated image $\hat{u}$ and the clear one *u*, and it is computed as:

(15) $$RMSE=\sqrt{\frac{1}{N.M}\sum_{x=1}^{N}\sum_{y=1}^{M}{\left(\hat{u}\left(x,y\right)-u\left(x,y\right)\right)}^{2}},$$

where *N* and *M* are the image dimensions. We used clear images and simulated the stripe non-uniformity with random fixed bias for each column. To do so, we generated a random normal distribution that have values between 0 and 1 with a standard deviation 0.05 and a size matching the number of columns, then each value from this distribution is added to one column as a fixed bias. The resulting images are then corrected using five different methods (BM3D [24] (sigma = 13), MIRE, IRUTV, GIF-based and the proposed). The corresponding PSNR results for each method are presented in Table 1. Results show a clear improvement when using the proposed algorithm in all the cases, which further proof the efficiency of the adopted approach.

## 4. Conclusions

In this work, an efficient method to correct for stripe non-uniformity while preserving edges and image textures is presented. The improved performance of the method comes from the introduction of a weighting factor that considers image structure during correction. The edge-aware weighting can efficiently describe edges and details of the image despite presence of noise. Additionally, a regularization process is also adopted for enhancing strong edges preservation ability. The comparison of the proposed algorithm with state-of-the-art de-striping techniques displayed its great efficiency in terms of noise reduction and edge-preserving.

## Figures and Tables

**Table 1:** The PSNR value under each correction method.

Images	1	2	3	4	5	6	7	8	9
noisy image	26.07	24.23	25.85	26.03	26.28	26.09	25.91	25.18	26.24
BM3D	29.86	27.45	27.47	29.87	30.61	31.11	29.23	29.12	30.31
MIRE	30.45	29.42	29.43	31.23	32.58	31.11	30.80	30.94	31.12
IRUTV	31.85	26.85	31.08	31.95	30.81	28.61	29.12	26.68	28.51
GIF_based	34.16	30.50	33.38	33.75	33.14	31.56	30.66	30.66	32.00
Proposed	**35.82**	**33.74**	**34.85**	**35.75**	**36.25**	**33.43**	**32.41**	**32.41**	**33.17**

## References

[1] Perry D.L., Dereniak E.L. (1993). Linear theory of nonuniformity correction in infrared staring sensors. Opt. Eng..

[2] Schulz M., Caldwell L. (1995). Nonuniformity correction and correctability of infrared focal plane arrays. Infrared Phys. Technol..

[3] Harris J.G., Chiang Y.M. (1999). Nonuniformity correction of infrared image sequences using the constant-statistics constraint. IEEE Trans. Image Process..

[4] Hardie R.C., Hayat M.M., Armstrong E., Yasuda B. (2000). Scene-based nonuniformity correction with video sequences and registration. Appl. Opt..

[5] Zuo C., Chen Q., Gu G., Sui X. (2011). Scene-based nonuniformity correction algorithm based on interframe registration. JOSA A.

[6] Cao Y., Yang M.Y., Tisse C.L. (2016). Effective Strip Noise Removal for Low-Textured Infrared Images Based on 1-D Guided Filtering. IEEE Trans. Circuits Syst. Video Technol..

[7] Tendero Y., Landeau S., Gilles J. (2012). Non-uniformity correction of infrared images by midway equalization. Image Process. Line.

[8] Cao Y., Li Y. (2015). Strip non-uniformity correction in uncooled long-wave infrared focal plane array based on noise source characterization. Opt. Commun..

[9] Chang Y., Yan L., Wu T., Zhong S. (2016). Remote sensing image stripe noise removal: From image decomposition perspective. IEEE Trans. Geosci. Remote Sens..

[10] Liu X., Lu X., Shen H., Yuan Q., Jiao Y., Zhang L. (2016). Stripe noise separation and removal in remote sensing images by consideration of the global sparsity and local variational properties. IEEE Trans. Geosci. Remote Sens..

[11] Münch B., Trtik P., Marone F., Stampanoni M. (2009). Stripe and ring artifact removal with combined wavelet—Fourier filtering. Opt. Express.

[12] Kuang X., Sui X., Chen Q., Gu G. (2017). Single infrared image stripe noise removal using deep convolutional networks. IEEE Photonics J..

[13] Zhao J., Zhou Q., Chen Y., Liu T., Feng H., Xu Z., Li Q. (2013). Single image stripe nonuniformity correction with gradient-constrained optimization model for infrared focal plane arrays. Opt. Commun..

[14] Chang Y., Fang H., Yan L., Liu H. (2013). Robust destriping method with unidirectional total variation and framelet regularization. Opt. Express.

[15] Huang Y., He C., Fang H., Wang X. (2016). Iteratively reweighted unidirectional variational model for stripe non-uniformity correction. Infrared Phys. Technol..

[16] Rudin L.I., Osher S., Fatemi E. (1992). Nonlinear total variation based noise removal algorithms. Phys. D Nonlinear Phenom..

[17] Goldstein T., Osher S. (2009). The Split Bregman Method for L1-Regularized Problems. SIAM J. Imaging Sci..

[18] Daubechies I., DeVore R., Fornasier M., Güntürk C.S. (2010). Iteratively reweighted least squares minimization for sparse recovery. Commun. Pure Appl. Math..

[19] Hua M., Bie X., Zhang M., Wang W. Edge-aware gradient domain optimization framework for image filtering by local propagation. Proceedings of the IEEE Conference on Computer Vision and Pattern Recognition.

[20] Kou F., Chen W., Wen C., Li Z. (2015). Gradient domain guided image filtering. IEEE Trans. Image Process..

[21] He K., Sun J., Tang X. (2013). Guided image filtering. IEEE Trans. Pattern Anal. Mach. Intell..

[22] Tomasi C., Manduchi R. Bilateral filtering for gray and color images. Proceedings of the Sixth International Conference on Computer Vision.

[23] Portmann J., Lynen S., Chli M., Siegwart R. People Detection and Tracking from Aerial Thermal Views. Proceedings of the IEEE International Conference on Robotics and Automation (ICRA).

[24] Dabov K., Foi A., Katkovnik V., Egiazarian K. (2007). Image denoising by sparse 3-D transform-domain collaborative filtering. IEEE Trans. Image Process..

